# Clinical and Genetic Findings of Turkish Hypophosphatasia Cases

**DOI:** 10.4274/jcrpe.4549

**Published:** 2017-09-01

**Authors:** Halil Sağlam, Şahin Erdöl, Sevil Dorum

**Affiliations:** 1 Uludağ University Faculty of Medicine, Department of Pediatrics, Division of Metabolism and Endocrinology, Bursa, Turkey; 2 Uludağ University Faculty of Medicine, Department of Pediatrics, Division of Metabolism, Bursa, Turkey

**Keywords:** Turkish children, Hypophosphatasia

## Abstract

**Objective:**

Hypophosphatasia (HPP) is a rare, commonly unrecognized hereditary mineralization defect with a dramatically poor prognosis in severe cases. This study is the first to examine the detailed clinical and laboratory characteristics of patients with HPP and healthy carriers in Turkey.

**Methods:**

The study data were obtained retrospectively from the files of 10 healthy carriers and of 16 cases with HPP (12 children and 4 adults) who were followed in our center from 2012 to 2016.

**Results:**

The annual incidence of perinatal lethal hypophosphatasia (PLH) was estimated to be approximately 1 case per 435,517 live births,, which is the first report from Turkey. The clinical courses of the cases differed depending on the type of HPP. All of the seven cases (58.3% of all cases) with perinatal lethal form of HPP died. A need for respiratory support (p=0.001), a history of pyridoxine-dependent seizures (p=0.001), a low chest circumference measurement (p=0.017), younger age at diagnosis (p=0.029), a small head circumference at the time of presentation (p=0.042), a low arm span to height ratio (p=0.048), and a low serum alkaline phosphatase (ALP) level (p=0.042) seemed to be predicting factors for mortality. The mean height standard deviation score of the patients and those of the healthy carriers did not differ significantly (p=0.173). Different mutations were detected in nine of 14 cases (64.2%) in whom an ALPL gene mutation analysis could be performed, and five of these cases (35.7%) had novel mutations. The most common mutations were c746G>T (five alleles), c346G>A (three alleles), and c.140C>T (three alleles). In addition, the most frequently observed genotype in Turkish HPP cases was autosomal-dominant c.346G>A (p.A116T) mutations which were detected in three cases in two different families.

**Conclusion:**

Because of the respiratory problems, especially the lung hypoplasia, the clinical course is poor in cases with the perinatal lethal form of HPP. Some minor abnormalities such as mild short stature and osteopenia could be observed in asymptomatic heterozygote carriers. Laboratory findings were normal in these cases.

**What is already known on this topic?**

Hypophosphatasia (HPP) is a bone mineralization disorder, commonly presenting with findings such as early loss of deciduous teeth and growth retardation. All HPP cases reported from Japan have an inherited autosomal recessive disorder.

**What this study adds?**

Some minor abnormalities such as mild short stature and osteopenia can be observed in asymptomatic heterozygote carriers whose laboratory findings are normal. Turkish HPP cases can exhibit an autosomal recessive or dominant inheritance similar to cases of European origin.

## INTRODUCTION

Hypophosphatasia (HPP) (OMIM# 241500, 241510, 146300) is a rare hereditary metabolic disease characterized by deficiency of alkaline phosphatase (ALP) due to mutations in the gene coding tissue non-specific ALP (TNSALP) enzyme (1p36.1-34) (1). ALP is a dephosphorylation enzyme that removes the phosphate group from many molecules, including nucleotides, proteins, and alkaloids. It is found in all tissues in the human body, most intensively in the liver, bile duct, kidneys, bones, small intestine mucosa, and placenta. TNSALP is the ALP originating from the liver, bones, and kidneys (2). TNSALP activity is found in bones, cartilage, and tooth tissue at almost all levels, and it is known to remove phosphorus from molecules containing phosphate, such as pyridoxal phosphate (PLP), phosphoethanolamine (PEA), and pyrophosphate (PPi). Bone mineralization is realized upon the placement of hydroxyapatite crystals composed of free calcium (Ca) and inorganic phosphate (Pi) in the collagen matrix. On the other hand, PPi is known as one of the most significant suppressors of hydroxyapatite formation. When TNSALP activity is low, PPi cannot be transformed into Pi, and as a result, serum PPi levels increase; the increasing PPi levels prevent bone and tooth development (2,3). Low TNSALP activity leads to low circulating pyridoxal levels within the central nervous system, negatively affecting γ-carboxyglutamic acid neurotransmitter synthesis and leading to pyridoxine-dependent seizures (4).

The ages at which the disease is initially observed cover a broad spectrum, from the fetal period to adulthood depending on enzyme activity, as is the case for almost all hereditary diseases. For this reason, the disease has been divided into the following clinical sub-groups according to age at presentation: perinatal lethal hypophosphatasia (PLH), prenatal benign hypophosphatasia (PBH), infantile hypophosphatasia, childhood hypophosphatasia, adulthood hypophosphatasia, and odontohypophosphatasia (odonto HPP). The ages at which the symptoms/findings appear and the severity of the disease are generally inversely proportional (2).

Cases may be identified through findings including insufficient mineralization in bones and teeth, early shedding of deciduous teeth, rickets, fractures, bone deformities, respiratory failure, growth and developmental retardation, hypotonia, pyridoxine-dependent seizures, hypercalcemia, hyperphosphatemia, nephrocalcinosis, craniosynostosis, and lower back pain, again depending on enzyme activity. When osteochondral formations called Bowdler projections (observed in the fibula and ulna) are detected, it can be deemed pathognomonic for the disease. Those formations can be observed in both fetal and benign perinatal forms (5).

It is estimated that the incidence of severe forms of the disease that affect both sexes equally is approximately 1 in 300.000 in Europe. Severe cases of the disease are generally inherited in an autosomal recessive manner, having a broad clinical spectrum, whereas inheritance varies in less severe cases (6).

The most significant key to the diagnosis is awareness of the disease. When HPP is brought into mind in the differential diagnosis of patients who present with bone, teeth, and other systemic symptoms and findings, it is very easy to diagnose the disease by establishing the low ALP levels (5).

An ALP replacement at bone level is required for treatment of cases with HPP. HPP has been recently added into the group of rare diseases that cannot be treated. Fortunately bone-directed recombinant human TNSALP (asfotase alfa) was developed and recently proved to be useful in a relatively broad group of patients with HPP (7).

In this study, we aimed to examine the clinical and laboratory characteristics of HPP cases and heterozygote carriers with normal laboratory findings, including TNSALP levels.

## METHODS

### Patients

The study data were obtained retrospectively from the files of 16 cases with a diagnosis of HPP and 10 healthy carrier cases that were followed in our center during the four years from 31 October 2012 to 1 November 2016. HPP cases whose age- and sex-matched serum ALP levels were low, who had no vitamin D insufficiency, and had not received bisphosphonate treatment, and healthy carrier family members were included in the study. The diagnosis was confirmed through an ALPL gene mutation analysis. Genetic analysis of the parents and siblings of the index cases was also performed. Healthy carriers, all of whom were parents or siblings of the HPP cases, were defined as those whose serum ALP, PEA, PLP levels were normal, did not have any complaints, and had a heterozygous mutation in the ALPL gene.

Detailed history and physical examination findings were obtained from the case files. The early loss of deciduous tooth and the existence of easy bone fractures were particularly questioned. Weight, height, head circumference, breast circumference from nipple and arm span were measured in all cases by the same specialist doctor using calibrated, standardized measurement tools. Arm span to length/height ratios were calculated and compared to the age- and sex-matched reference values (8). All of the comparisons of the anthropometric measurements were evaluated according to the age- and sex-matched centiles and/or standard deviation score (SDS). The data on number of teeth were obtained from the file. All of the cases were assessed at baseline and at each visit in terms of the tooth status which is defined as the number of missing teeth compared to the number they should actually have according to his/her age. A neurological examination was performed by the same child development specialist by using the Bayley developmental motor scale test in cases up to 42 months old and by using the Peabody developmental motor scale test in cases from 42 months to 18 years old. The motor development of adults was evaluated by an adult neurologist. Gross motor development, which was expected to be mostly affected, was evaluated in the comparisons.

Serum Ca, P, ALP, PTH and vitamin D levels, urine Ca/Cr ratios, bone radiographs, lumber region dual-energy X-ray absorptiometry (DEXA) measurements, ALPL gene mutation analysis, urine PEA levels, and plasma PLP values were obtained from the medical records. In DEXA assessments, T-scores in adults and Z-scores in children were considered. A detailed eye examination was performed to identify any papilledema or calcifications, and a renal ultrasound was performed to identify the existence of nephrocalcinosis in all cases by the same pediatric ophthalmologist and radiologist.

### Classification of the Patients

Cases whose findings were evident in the intrauterine and newborn periods and whose clinical course was poor were classified as those with the perinatal lethal form of the disease, and those whose clinical course was good were classified as being afflicted with the prenatal benign form. Those who revealed symptoms between 1 and 6 months were classified as infantile HPP. Cases whose symptoms became apparent between 6 months and 18 years were classified as childhood, and after the age of 18 years were classified as adulthood HPP. Cases whose course included only a tooth finding in both childhood and adulthood were classified as odonto HPP (9).

### Radiology

All of the radiologic studies were performed in our center. Bone mineralization data of the cases were determined using both conventional X-ray radiographies and DEXA. Conventional X-ray radiographs were studied with the Xgeo GC80 (Samsung Electronics Co. Ltd., Suwon, South Korea) device and DEXA was studied with a horizon (Hologic, Inc., Bedford, MA, USA) device. Anterior-posterior skull and chest x-rays, and bilateral upper and lower extremity radiographs were taken using conventional X-ray radiography. Lumbar L1–L4 vertebral bone mineral densities were measured using DEXA. All the assessments were performed by the same specialist radiologist and by the authors.

### TNSALP Gene Mutation Analysis

PCR products were purified and sequenced using the direct sequencing method and the ABI PRISM Dye Terminator Cycle Sequencing Ready Reaction kit with AmpliTaq DNA polymerase, FS (Perkin-Elmer Corp., Foster City, CA, USA), and they were moved to an ABI PRISM 310 electrophoresis system (Applied Biosystems, USA). The method of Mornet et al (10) was used in the PCR amplification of exons and single strand conformation polymorphism analysis.

### Statistical Analysis

The compliance of the data included in the study with a normal distribution was assessed using the Shapiro-Wilk test. Data not in compliance with a normal distribution were compared using the Mann-Whitney U test, and their descriptive statistics were expressed as the median (minimum-maximum). A Pearson chi-square test was used to compare the categorical variables between groups. The IBM SPSS Statistics 23 software package was used for statistical analysis. In comparisons, p-value <0.05 was considered statistically significant.

## RESULTS

During the study period of 4 years, due to our involvement with an international HPP study, all cases diagnosed as having HPP throughout Turkey were being directed or referred to our center. The number of PLH cases referred to us over the four years is seven, and in addition, five other PLH cases in other cities died before reaching our center. Thus, the number of PLH cases diagnosed in Turkey within this period of four years is 12, as far as we could detect. The total number of newborn babies in Turkey from 2012 to 2016 is 5,226,213 according to the data of the Turkish Statistical Institute (11). Consequently, the incidence of PLH in our country is estimated to be approximately 1 case per 435,517 live births. The estimated annual number of PLH cases is three. It was not possible to estimate an annual incidence for cases with other HPP forms which could easily go unrecognized because of the relatively subtle findings.

All 16 HPP and 10 healthy carrier cases participating in the study were of Turkish ethnic origin and were directed to our center from various provinces of Turkey from 2012 to 2016 with a suspicion of HPP.

Of the HPP cases, seven (43.8%) were PLH, three (18.7%) PBH, three (18.7%) odonto HPP, two were (12.5%) adult HPP, and one (6.3%) was childhood HPP. The clinical and laboratory characteristics of those cases are summarized in Table 1. The median follow-up duration of all the patients was 1 year (min=0.04 years; max=3.66 years).

Median age at HPP diagnosis was 0.58 years (range, 0-35 years), whereas median age of heterozygote cases whose laboratory characteristics were normal was 8.15 (range, 6-55) years.

In total, 43.8% (n=7) of HPP cases were female and 56.2% (n=9) were male. This proportion was similar in carrier cases as well (40% vs. 60%). The male gender predominated in PLH (71.4%), but no statistically significant difference was detected in terms of gender between HPP cases and their heterozygous family members with normal laboratory findings (p=0.85).

Clinical findings differed depending on the form of HPP. The most frequently observed findings were short stature, short extremities, epilepsy, large fontanel, motor retardation, and respiratory failure (Table 2).

At presentation, no statistically significant difference was detected between the height SDS of HPP cases and those of carrier cases with normal laboratory values (-2.06 vs. -1.62; p=0.173). Regarding height and arm span/height ratios, mean arm span/height ratio in HPP cases at presentation was 0.94, and this ratio was found to be low compared to normal reference data in all cases whose age at diagnosis was below three years (n=10). As well, this proportion was found to be normal in all patients diagnosed after age three years with a mild clinical course (n=6). The average arm span/height proportion of carrier cases not having HPP (n=10) was 0.98, and all were within the normal limits (Table 3) (8).

During the follow-up interval of about 4 years, no change was observed in terms of rough motor functions in 43.8% (n=7) of cases, whereas 37.5% (n=6) had deterioration and 18.8% (n=3) had improvement. Gross motor functions of all healthy carrier cases were normal.

None of the heterozygote carriers not having HPP experienced early loss of deciduous teeth; while six of nine HPP cases (66.6%) whose teeth had erupted, experienced early loss of deciduous teeth.

The tooth status of four (44.4%) of nine cases with HPP whose teeth had erupted worsened when the basal and final assessments were considered, while the tooth status of the 10 heterozygote carriers not having HPP did not change.

Conventional radiography revealed osteopenia in 10 (62.5%) and rickets in 7 (43.7%) of the HPP cases. All of the cases having rickets also had osteopenia. Rickets or osteopenia were found in none of the carriers not having HPP (Table 2).

DEXA T-/Z-scores of cases having HPP were found to be lower than those of healthy carriers, but the difference was not statistically significant [-1.7 (range, -3.1-0.1) vs. -0.9 (range, -1.1- -0.9)], (p=0.381).

Survival significantly differed among HPP forms (p=0.001). All cases who succumbed were of PLH form and all PLH cases died. Moreover, a statistically significant difference was found between cases who survived and cases who died with respect to age at diagnosis (p=0.029), head circumference at the time of presentation (p=0.042), arm span to height ratio (p=0.048), chest circumference (p=0.017), and serum ALP level (p=0.042). The chest circumferences of all of the seven cases who died were <3 percentile, and the chest circumference of only one (11.1%) of the nine living HPP cases was <3 percentile. Serum ALP levels of PLH cases, who all died, and of PBH cases, who all survived, were 10.0 IU/L (range, 6.0-38.0) and 31.0 IU/L (range, 24.0-45.0), respectively. Major clinical and laboratory findings of the HPP forms are given in Table 4.

One of the most significant factors related to survival was the need for respiratory support (p<0.001). Respiratory support was needed in all of the seven cases who died, while none of the nine living cases needed respiratory support. While all PLH cases had respiratory failure, respiratory assessment showed normal function in all PBH cases. 

Moreover, there was a significant difference between patients who survived and those who died with respect to existence of pyridoxine-dependent seizures (p=0.001). While all seven cases who died had pyridoxine-dependent seizures, there was no history of pyridoxine-dependent seizures in any of the nine surviving cases.

### TNSALP Mutation Analysis

Genetic analysis was available for 14 of 16 HPP cases. Of these, 9 (64.2%) had different mutations and five (35.7%) were novel disease-causing mutations according to the Mutation Taster pathogenicity prediction program. The c746G>T (five alleles), c346G>A (three alleles), and c.140C>T (three alleles) were the most frequently observed mutations. The most frequently observed genotype in HPP cases was the autosomal dominant c.346G>A (p.A116T) heterozygote mutation, detected in three cases in two different families. Five of eight cases (62.5%) having a homozygote mutation had PLH, whereas none of the heterozygote cases had PLH (Table 1).

## DISCUSSION

The clinical and genetic characteristics of Turkish HPP cases were specified with this retrospective study. No data on HPP incidence in Turkey are available, and it differs across the world. The data on incidence are more often related to PLH as mild forms could be missed. The incidence of PLH in Japan is 2-3 in 1 million live births annually (12), whereas this value was estimated to be 1 in 100,000 in Canada (13). The annual PLH incidence in our country was found to be approximately 3.06 cases per 1 million live births in this study. PLH is the form most frequently observed (43.7%) in the patients referred to our center, similar to the data in Japan (12), and this form is observed more frequently in males (71.4%).

Bone deformities were detected in fetal ultrasonography (USG) of two PLH and two PBH cases included in the study, and fetal USG findings in both forms were similar. In line with previous literature data, these results support the argument that the findings obtained through fetal USG, do not predict the clinical course.

Since the ratio of severe clinical forms was high among our HPP cases, clinical findings were more pronounced and more common in most of the cases. For example, the incidence of pyridoxine-dependent seizures was observed at a rate of 42.8% in Japanese PLH cases, whereas pyridoxine-dependent seizures were detected in all of the Turkish PLH cases followed in our center. Similarly, in a study conducted by Taketani et al (12), the ratio of short stature in cases with HPP was 42.8%, a ratio higher than that reported from western countries. This figure was even higher (75.0%) in our patients. Also, three of four cases without short stature were odonto HPP cases and one was an 8-year-old PBH case.

The most significant prognostic factors detected in our HPP cases were respiratory failure and pyridoxine-dependent seizures, both indicating a worse prognosis and this finding conformed with previous studies (2,5). In addition, chest circumference, age at diagnosis, head circumference, arm span to height ratio, and serum ALP levels were found to be predictors of prognosis in our HPP cases. In other words, as the measurements of chest circumference, age at diagnosis, head circumference, arm span to height ratio, and serum ALP levels decrease, the prognosis worsens.

To our knowledge, heterozygote family members of HPP cases with normal laboratory values have not been studied so far. Our attention has been drawn to the fact that the serum ALP levels of heterozygote family members of HPP cases were generally normal but within the lower third of the sex-matched adult reference values, and this could have led to some mild abnormalities. For example, the mean height SDS of the carrier family members without HPP was -1.62 which was similar to that of HPP cases (-2.06). Similarly, the median T-/Z-score of carrier heterozygote cases was -0.9 which is not statistically different from that of the HPP cases (-1.7) (Table 3). These findings may indicate that the carriers have slightly low height and low bone mineral density compared to the mean of population, although they are healthy.

Certain mutations can lead to severe HPP forms in some societies, such as the existence of a homozygote 1559delT mutation in the ALPL gene leading to PLH in a Japanese society (12). However, this was not the case in the present study as the mutations of all Turkish PLH cases were different.

Mutations observed in the ALPL gene could vary between societies; for example, the mutations among all the reported Japanese HPP cases were inherited in an autosomal recessive manner (12). The mutations among the Turkish HPP cases could exhibit an autosomal recessive or dominant heritage, similar to cases of European origin. An autosomal recessive heritage leads to HPP forms that are more serious, whereas an autosomal dominant heritage leads to more minor forms, such as odonto HPP and childhood HPP. Because the family members of the HPP cases were assessed through detailed physical, laboratory, and radiologic examinations in this study, some mild HPP forms were detected in the mothers of cases with severe forms of HPP who have a homozygote mutation. For example, the case having the novel c.140C>T (p.N47I) homozygote genotype had PLH, whereas her heterozygote mother had adult-type HPP. Moreover, the case having the c.746G>T (p.G249V) homozygote genotype had clinical PBH, while his heterozygote mother had clinical odonto HPP. Interestingly, fathers of both patients having the same heterozygous mutations were completely normal. Though most of the heterozygote cases in many other inherited metabolic diseases do not reveal clinical phenotype, this may not be the case in HPP.

A genotype-phenotype correlation for HPPs except PLH has not been suggested so far in previous studies. For example, HPP of different clinical severity was observed in two siblings having the same mutation (12). In our study, we also observed similar findings. Two siblings having PBH with the c.746G>T (p.G249V) homozygote mutation exhibited a different clinical course. Although both had highly elevated serum PLP and urinary PEA levels, the boy exhibited a mild phenotype and all his clinical findings spontaneously improved at age 9, while the girl showed more pronounced clinical findings and was still having abnormal clinical findings at the age of 2.5 years. On the other hand, it has been suggested that the 1559delT mutation, which is only observed in Japanese cases, could lead to PLH (14).

The three mutations detected in our study led to a different clinical course compared to the cases with the same mutations in the literature. For instance, while the c.815G>A (p.A272H) homozygote mutation in the literature led to infantile HPP (15), it led to PBH in our case. Similarly, while the c.746G>T (p.G249V) homozygote mutation in the literature was suggested to lead to PLH (16), it led to PBH in our two sibling cases, and again, though the c.346G>A (p.A116T) heterozygote mutation in the literature was related to odonto HPP (17), it led to both childhood and odonto HPP in our study (Table 1). This suggests that the same mutation may lead to different clinical outcomes in different cases.

As expected, clinical HPP forms having a homozygote mutation in the ALPL gene generally have a more serious clinical outcome and the clinical findings appear early compared to heterozygote cases. Interestingly, in this peculiar disease, some cases with a homozygote mutation in the ALPL gene exhibiting very striking clinical findings at an early age, even in the intrauterine period, may have a very favorable clinical outcome, as is the case in most of the PBH cases.

In conclusion, in this first HPP study from Turkey, annual PLH incidence was estimated to be approximately 1 per 435,517 live births. PLH was the most frequently observed form. The most frequently observed findings were short stature, short extremities, epilepsy, large fontanel, motor retardation, and respiratory failure. The most significant predictors of poor prognosis in HPP cases are pyridoxine-dependent seizures and respiratory failure mainly caused by a hypoplastic thorax. Although intrauterine bone anomalies may indicate HPP, they fail to predict the course of the disease. Mild short stature and osteopenia can be observed in asymptomatic heterozygote cases whose laboratory findings are normal. Both autosomal recessive and dominant mutations can cause HPP phenotype in the Turkish population.

## Figures and Tables

**Table 1:**
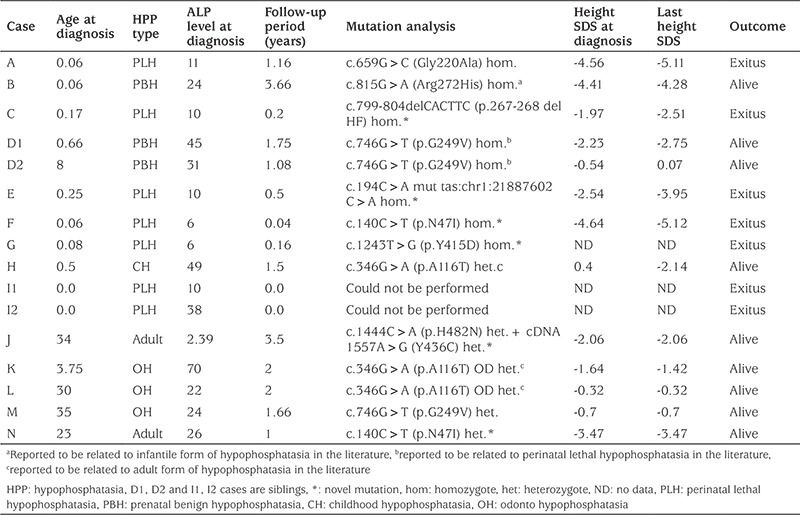
Clinical and laboratory characteristics of cases with hypophosphatasia

**Table 2:**
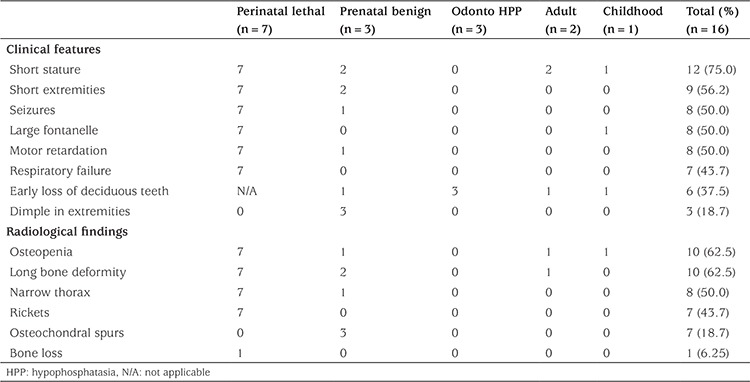
Clinical and radiological characteristics of hypophosphatasia cases

**Table 3:**
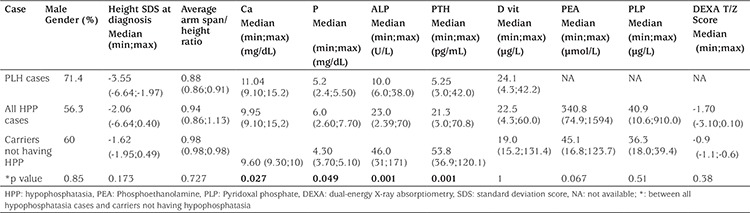
Comparison of clinical and laboratory characteristics of cases with hypophosphatasia and of carriers not having hypophosphatasia

**Table 4:**
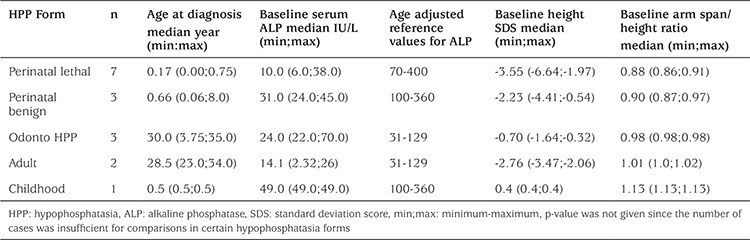
Principal clinical and laboratory characteristics by hypophosphatasia forms
